# Associations Between Asthma and Polycystic Ovary Syndrome: Current Perspectives

**DOI:** 10.3389/fendo.2022.936948

**Published:** 2022-07-05

**Authors:** Yue Xu, Zhi-Yang Zhou, Jie-Xue Pan, He-Feng Huang

**Affiliations:** ^1^ Obstetrics & Gynecology Hospital, Institute of Reproduction and Development, Fudan University, Shanghai, China; ^2^ Shanghai Key Laboratory of Embryo Original Diseases, International Peace Maternity and Child Health Hospital, School of Medicine, Shanghai Jiao Tong University, Shanghai, China; ^3^ Research Units of Embryo Original Diseases, Chinese Academy of Medical Sciences, Shanghai, China

**Keywords:** polycystic ovary syndrome, asthma, metabolic syndrome, chronic inflammation, reproductive health

## Abstract

A potential correlation between polycystic ovary syndrome (PCOS) and asthma, used to be identified as diseases originating from two independent systems, has been supported by increasing evidence. From an epidemiological perspective, mounting studies have confirmed that women suffering from PCOS exhibit increased susceptibility to asthma. Meanwhile, PCOS and asthma seem to share several mutual pathological conditions, such as metabolic disorders, hormonal fluctuation, proinflammatory state, etc. Here, we further elucidate the correlation between asthma and PCOS by focusing on the internal common pathophysiology and adverse influences on women’s health. Understanding the internal connection between PCOS and asthma may shed light on developing new prevention and control strategies to fight against these conditions.

## Introduction

As a common endocrinopathy among women of childbearing age, polycystic ovary syndrome (PCOS) poses a considerable threat to the health of women throughout the whole world with a prevalence of 15%-20% (1, 2). According to the revised 2003 Rotterdam criteria, a final diagnosis of PCOS can be made if two of the following indicators are met: hyperandrogenism, ovulatory dysfunction, and polycystic changes in the ovary (3). In addition to reproductive health issues (4), PCOS carries an important responsibility for female metabolic disorders and mental problems (5, 6). Notably, chronic systemic and local ovarian inflammation has emerged as a vital focus for the study of PCOS pathophysiology (7–9). To be more specific, previous studies have indicated that PCOS women usually exhibit abnormally elevated levels of inflammatory cytokines (e.g. TNF-α, interleukins, CRP) (9), as well as DNA single-nucleotide polymorphisms of relevant inflammatory mediators (10).

Asthma, a chronic airway inflammatory disease with recurrence and reversibility, is characterized by paroxysmal wheezing, shortness of breath, chest tightness, and cough. To date, approximately 235 million people worldwide suffer from asthma, causing a considerable burden of disease to both families and societies (11). The pathogenesis of asthma involves complex interplay of multiple factors, such as airway hyperresponsiveness (AHR), chronic low-grade inflammation, and airway structural changes (12). In particular, intensive investigations have proposed a potential causal relationship between hormonal fluctuation and the occurrence or severity of asthma, and extensively highlighted the gender disparities that can change with age, which probably accounts for the reproductive disorders in asthmatic women. Intriguingly, as indicated by epidemiological studies, females with metabolic syndrome tend to exhibit an elevation in susceptibility to asthma attacks, whereas the mechanisms underlying the pathophysiology are poorly understood.

**Figure 1:**
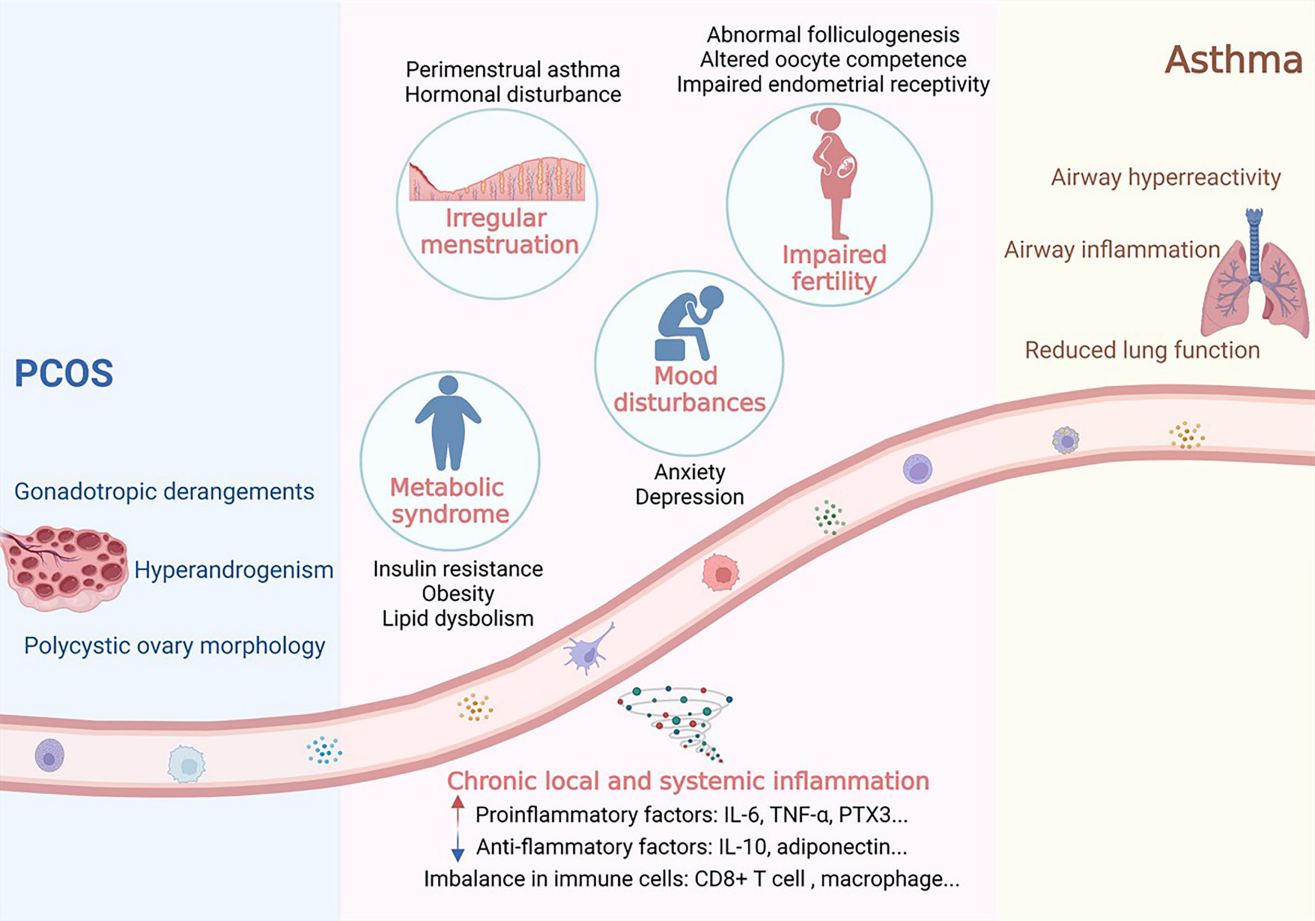
Potential associations between PCOS and asthma. Women with PCOS or asthma are characterized by several common disease phenotypes, including metabolic syndrome, irregular menstruation, impaired fertility and mood disturbances. And chronic local and systemic inflammation may act as one of the common molecular mechanisms behind these phenomena. Image was created with Biorender^®^.

Following the similar risk factors as well as the intersection of potential pathogenesis, several lines of evidence have gradually revealed a non-negligible association between asthma and PCOS in metabolic syndrome, impaired fertility, irregular menstruation, and mood disturbances. And chronic local and systemic inflammation may act as one of the common molecular mechanisms behind these phenomena. Therefore, by literature retrieval and inductive analysis, we further expounded the correlation between asthma and PCOS by focusing on the internal common pathophysiology and insults on female health, to provide unique insights for further exploration of disease prevention and therapy (**Figure 1**).

## Concomitance of Asthma and PCOS

PCOS is comorbid with respiratory diseases with a considerable frequency, and asthma in particular. A retrospective cohort study in 2015, which collected and mined the data from Australian statewide hospitals, reported that the prevalence of respiratory disease in women with PCOS was about 22.8%, compared with 14.2% in the control group; among them, 10.6% of the former were admitted to hospital for asthma, while only 4.5% of the latter, suggesting that women with PCOS were more prone to develop asthma (13). And other epidemiological studies of different populations have reached similar conclusions (4, 12–16). In support of the above findings, Underdal et al. propose a potential link between PCOS and asthma from the perspective of lung function, reporting a combined obstructive (forced expiratory volume in one second (FEV1) % predicted, 93.7 vs 102.0) and restrictive (forced vital capacity (FVC) % predicted, 94.5 vs 103.7) respiratory impairment in PCOS compared with controls (17). Meanwhile, a historical cohort study reported that irregular period was associated with higher odds of atopic asthma (18), which once again verified the potential connection between asthma and ovulatory dysfunction. Recent studies have further explored whether confounding factors alter the association between asthma and PCOS, and indicate that the incidence of asthma remained higher even after adjusting for heterogeneity of body mass index (BMI), smoking status, and dietary intake (12, 16). Furthermore, Sun H et al. (19) performed a meta-analysis that systematically evaluated the prevalence of asthma as related to PCOS, and determined PCOS as an independent risk factor for asthma.

Although the link between PCOS and asthma incidence is well established, results from different studies on asthma severity among women with or without PCOS are conflicting. To be specific, Zierau et al. investigated the severity of asthma based on the use of anti-asthma medication, and indicated that PCOS status is not a contributing factor to exacerbation of asthma (14). However, other studies report opposite results, which are verified by a higher hospital admission rate for asthma and increased doses of anti-asthma medication in PCOS women (13, 15). In combination, the researches mentioned above suggest an important correlation between PCOS and asthma attack and provide a reliable perspective to investigate the corresponding pathogenesis (**Table 1**).

**Table 1:** General characteristics of key studies.

Author	Year	Country	Study design	PCOS criteria	Asthma criteria	Characteristics of the subjects		Asthma incidence % (n)		P value
						PCOS	Control	PCOS	Control	
Hart et al. (13)	2015	Australia	Retrospective cohort study	ICD-10	Medical record	Median age: 27.9	Not available	10.56% (271/2566)	4,52% (1160/25660)	<0.01
Underdal et al. (17)	2020	Norway	Prospective cohort study	Rotterdam criteria	Medical record	Mean age: 38 Mean BMI: 28.5	Mean age: 38 Mean BMI: 25.7	19.31% (28/145)	8.74% (38/435)	<0.01
Doherty et al. (4)	2015	Australia	Retrospective cohort study	ICD-10	Medical record	Not available	Not available	13.64% (478/3505)	9.90% (3450/34856)	<0.01
Zierau et al. (14)	2018	Denmark	Prospective cohort study	Rotterdam criteria	Medical record	Mean age: 34 BMI: 25.8 ± 5.3	Mean age: 34 BMI: 24.3 ± 4.9	19.59% (266/1358)	14.48% (786/5430)	<0.001
Glintborg et al. (15)	2015	Denmark	Prospective cohort study	ICD-10	Medical record	Mean age: 30.6	Mean Age: 30.6	3,05% (586/19199)	2.21% (1270/57483)	<0.001
Htet et al. (16)	2017	Australia	Prospective cohort study	Questionnaire	Questionnaire	Age: 30.5 ± 0.1 BMI: 28.0 ± 0.3	Age: 30.6 ± 0.02 BMI: 25.1 ± 0.1	16.11% (77/478)	10.52% (856/8134)	0.004
Grieger et al. (12)	2020	Australia	Prospective cohort study	Questionnaire	Questionnaire	Age: 33.5 ± 1.4 BMI: 28.5 ± 7.4	Age: 33.7 ± 1.5 BMI: 25.6 ± 5.8	13.64% (478/3505)	9.90% (3450/34856)	0.004

## Overlap Between Features of PCOS and Asthma

### Metabolic Syndrome

Metabolic syndrome (MS) is a cluster of complex disorders characterized by abnormal glucose metabolism, dyslipidemia, overweight or obesity, and hypertension, and has been recognized as the most critical long-term health problem associated with PCOS. Both obese and lean women with PCOS are reported to have a much higher risk of MS compared with the control group (20–22). So far, numerous pathologic changes have been identified in exploring the pathogenesis of PCOS, such as hyperandrogenemia, hyperinsulinemia, dysfunction of the hypothalamic-pituitary-ovarian axis, and disturbances of adipokines secretion. These specific alterations interact with each other in fat tissues, liver, muscle, and ovary, resulting in diverse phenotypes of MS.

Remarkably, insulin resistance (IR) and hyperandrogenism stand out as pivotal factors in the development of PCOS. Approximately 50-80% of PCOS females exhibit varying degrees of IR, which possibly accounts for the higher annual conversion rate to impaired glucose tolerance (IGT) or diabetes mellitus type 2 (T2DM) in them (23, 24). Previous studies have identified several abnormal or dysfunctional cells and molecules involved in the IR status of PCOS women, such as glucose transporter 4 (GLUT4), insulin receptor β subunit, pancreatic β-cells, and so on (25, 26). In PCOS, adipose tissue dysfunction, possibly leading to excessive visceral fat depots, is thought to be involved in determining both IR and dysregulated androgen metabolism (27). When exposed to excessive androgen, adipocytes seem to be prone to hypertrophy, releasing superabundant adipokines and inflammatory mediators. TNF-α, for instance, can block the tyrosine kinase phosphorylation of insulin receptors (28), and affect glucose transport by inhibiting GLUT-4 activity (29), thus inducing the obesity-related IR or hyperinsulinemia. Functionally, insulin acts as an essential factor in the development of hyperandrogenism (30). Insulin not only directly stimulates follicular theca cells to produce androgen and encourages the development of stromal cells in the ovary, but also induces the secretion of insulin-like growth factor 1 (IGF-1) so as to promote androgen synthesis in adrenal and gonads (31). Thus, adipose tissue dysfunction, inflammation, IR, and hyperandrogenism form a vicious cycle that leads to different phenotypes in PCOS patients.

In recent years, growing evidence also supports MS is a potential risk factor for asthma. Multiple studies have linked MS to asthma attacks but the correlation is still under dispute. Lee et al. (32) put forward that patients with MS are more likely to develop symptoms of wheeze and dyspnea at rest or after exercise. This is consistent with a large retrospective cohort study from Norway showing that excessive waist circumference and diabetes increase the susceptibility to asthma in adults (33). What’s more, cohort studies in Japan and China also revealed that overweight or obesity can dramatically aggrandize susceptibility to asthma in females but not in males (21, 34). The sexual heterogeneity makes it reasonable to presume that specific gonadal hormones and metabolic disorders may complement each other to exert promoting effect on the pathogenesis of asthma. However, whether every element of MS can induce female asthma attacks is still controversial. Assad et al. pointed out that the association between abdominal adiposity, elevated blood pressure, impaired fasting glucose or diabetes and asthma incidence is no longer statistically significant after adjustment for BMI (35). This raises the possibility that BMI or obesity is a stronger predictor of asthma than MS. In turn, so far little is known about the influence of asthma on obesity incidence. Several studies have shown that early-life of asthma may result in a higher susceptibility to obesity in later childhood and adolescence (36, 37), but further investigations are needed to verify the promoting effect of asthma on obesity.

The underlying mechanism of overweight or obesity prompting asthma remains uncertain, and attention is mainly focused on the mechanical factors and altered inflammation responses. First, obesity is theorized to encourage drastic changes in normal lung physiology. Excessive accumulation of fat in the chest and abdominal cavity contributes to lung compression and reduced lung volume, resulting in a pronounced decrease in functional residual capacity (FRC) and expiratory volume (ERV) (38, 39). Low lung volume leads to AHR through impaired ability to stretch the airway wall (40). Among patients with late-onset non-allergic asthma, obesity induces excessive collapsibility of airways by reducing distal airway wall stiffness (41). Second, it is not unexpected that obesogenic dietary patterns tend to contain much fatty acids and fructose but little fiber and antioxidants. Growing evidence verifies that food rich in saturated fatty acids can encourage pulmonary lymphocyte infiltration, induce congenital AHR and further allergic airway inflammation through the IL-1β pathway (42, 43). Third, inflammatory effects of obesity on the lung involve dysregulation of proinflammatory adipokines (e.g. excess leptin) and anti-inflammatory adipokines (e.g. adiponectin deficiency). Excessive adipose tissue secrets redundant pro-inflammatory cytokines, such as TNF-α, interleukin, and leptin, which further trigger systemic inflammation and AHR. Meanwhile, the downregulated level of bronchoalveolar surface-active protein A (SPA) in obese subjects with asthma can cause airway eosinophil aggregation (44), thereby increasing the risk of allergic airway inflammation. In combination, MS may then act as a link between PCOS and asthma, with a co-existence of MS and PCOS aggravating asthma.

### Impaired Fertility

It is well established that PCOS status threatens fertility in women of childbearing age. Although women with PCOS are typically characterized by an increase in the quantity of preantral follicles, the quality of oocytes is usually diminished, resulting in impaired fertilization, cleavage as well as embryo implantation. Wild et al. (45) demonstrated that 17.5% of PCOS women, 11 times that of the control group, reported fertility problems, of which 66% of PCOS women suffered from infertility and 24% relied on ovulation induction. Consistently, other researchers also observed remarkable growths in the non-fertility rate, gestational age, and the utilization rate of assisted reproductive technology in PCOS women (46, 47). Hormonal imbalance is the most striking feature of PCOS, accounting for diverse reproductive problems. In PCOS, hypersecretion of LH during follicle formation significantly impairs oocyte maturation, fertilization, and embryo quality (48, 49). Part of the cellular and molecular abnormalities caused by excessive LH have been identified, including suppressed FSH function, dysfunctional granulosa cell (GC), damaged oocyte nucleus, and impaired extrusion of the first polar body (50, 51). Excessive insulin can cooperate with LH to stimulate androgen production (52), which thereby retards the development of dominant follicles, impacts endometrial receptivity, and increases the risk of various obstetric complications (53–55).

Recently, much more attention has been paid to the similar association between allergic diseases and infertility. Gade et al. (56) revealed that asthma was responsible for a prolonged time to pregnancy (TTP), with TTP of about 27% of asthmatic women extended beyond one year, and specific treatment for asthma could effectively abolish the extension of TTP (57). Hansen et al. (58) explored the association between asthma and the need for fertility treatment among women with life births, and pointed out that the association remained significant after adjusting for age, BMI, and smoking habits. What’s worse, despite fertility treatment, asthma is still associated with unfavorable pregnancy outcomes (59). Rocklin et al. reported that maternal asthma may increase the risk of preterm birth, low birth weight, and perinatal mortality (60). Overall, the vast majority of evidence points toward the possibility that asthma in women has an inextricable link with delayed conception, lowered genitality, and poor pregnancy outcomes.

Obesity and inflammation response are two pathologic conditions closely related to both PCOS and asthma, which may provide insights into the impaired fertility associated with these two conditions. Obesity is partially responsible for poor oocyte quality through a variety of pathways, including follicular development retardation, oocyte maturation problems, meiosis abnormalities, as well as mitochondrial dynamics disorder (61–64). Excessive free fatty acids are presumed to exert toxic effects on reproductive tissues through oxidative stress, leading to damage or apoptosis of female gametes (64). Recently, studies have demonstrated that several adipokines may carry metabolic responsibilities for female reproductive function, especially in ovarian physiology. Through the interaction with LH and insulin, adiponectin encourages the expression of genes related to periovulatory maturation of ovarian follicles (65). Lower adiponectin levels were reported in the plasma of either PCOS (66) or asthma (67) women, as well as in the follicles of PCOS women (68). These suggest its possible role in the follicular arrest and ovulatory dysfunction. In combination, obesity, independently or jointly with endocrine disruption, impair female fertility in women with PCOS or asthma.

What’s more, abundant available evidence supports chronic systematic inflammation as the common cornerstone for the internal pathogenesis of impaired fertility in both PCOS and asthma. The immune system plays a pivotal part in the reproductive process. Concretely speaking, Th2 immunity supports the pregnancy process by reducing the rejection of fetal tissue, while Th1 immunity is thought to be unfavorable to the “foreign” fetus (56). Based on this recognition, researchers speculate that asthma might cause female fertility impairment and poor pregnancy outcomes by inducing an imbalance of the adaptive immune system. The systemic inflammation induced by asthma may increase the infiltration of inflammatory cells and alter cytokine profiles in the ovary and uterus exerting a negative effect on fertility. For example, TNF-α is regarded as a potential regulator of follicular development, ovulation, and oocyte apoptosis, playing an important role in reproductive failure (69–71). Imbalanced IL-6 is associated with recurrent miscarriages and failed implantation by regulating the proliferation, differentiation, and survival of germ cells (56, 72). Intriguingly, growing evidence supports that PCOS is also closely related to chronic systemic inflammation. The amount of immune cells in the peripheral blood of PCOS women dramatically increases with high androgen levels, inducing the secretion of inflammatory factors such as CRP, TNF-α, and IL-6 (73). In addition, previous studies revealed the altered immune cell profiles (e.g. macrophages, dendritic cells, and CD8^+^ T cells) in the ovary and endometrium of PCOS women (74, 75), which possibly compromise ovarian function, normal implantation, and endometrial health in the long run. For example, pentraxin-3 (PTX3), a crucial humoral innate immunity component secreted by macrophages, which stands out as a structural constituent of the cumulus oophorus extracellular matrix essential for female fertility, was reported abnormally high both in plasma and ovary in PCOS women (76–78). And higher PTX3 is associated with hyperandrogenism (79), which might perturb interactions of the sperm with the matrix during *in vivo* fertilization (80). Low-grade systemic inflammation, therefore, carries pivotal responsibility for impaired female fertility, which may build a bridge connecting asthma and PCOS.

### Irregular Menstruation

Irregular menstruation refers to oligomenorrhea, amenorrhea, abnormal uterine bleeding, etc. Numerous studies have revealed that PCOS women tend to exhibit a higher prevalence of irregular menstruation, which is often initially onset at a younger age and is positively correlated to BMI (81, 82). Compared with the age-matched controls, PCOS females reported more frequent hospitalization for menstrual abnormalities (20.3% vs 4.9%), endometriosis (26.4% vs 4.4%), endometrial hyperplasia (1.8% vs 0.1%) and other gynecological diseases (13). Based on the available evidence, lifestyle interventions, insulin sensitizers, and anti-androgen drugs are thought to be able to improve the reproductive health of PCOS women by inhibiting IR or declining androgen levels.

Abnormal regulation of steroidogenesis and ovarian regulation is a pivotal part of the pathophysiology of PCOS. Hypersecretion of LH and IGF-1, anti-Müllerian hormone (AMH) interact with each other to induce hyperandrogenism (83). For one thing, excessive androgen enhances the initial recruitment of primordial follicles into the growth pool, thus regulating the growth of small antral follicles (84). For another, hypersecretion of androgen encourages premature luteinization, thus inhibiting the selection of the dominant follicle and hindering normal ovulation (49, 85). Ovarian follicular arrest results in oligo-ovulation or anovulation, characterized by delayed menstruation, amenorrhea, or abnormal uterine bleeding (85).

Meanwhile, growing evidence demonstrates a strong correlation between menstruation and asthma attacks. Earlier menarche or irregular menstruation seems to predict worse lung function and increased susceptibility to asthma in adulthood (86–88). Women with abnormal or irregular menstrual cycles tend to exhibit significantly lowered FVC and elevated prevalence of asthma attacks (89). Furthermore, the exacerbation of asthma during menstruation, known as perimenstrual asthma (PMA), affects 30-40% of women with asthma. Compared with normal asthma attacks, PMA is characterized by more severity, increased drug use, lower vital capacity and PEF, as well as higher airway responsiveness (90). The association between deviations in levels of sex hormones and PMA has been suggested. Compared to non-PMA asthmatics, PMA subjects exhibit elevated estradiol levels in the luteal phase of the cycle (91). Rubio et al. reported that on the 5th and 21st days of the menstrual cycle, approximately 80% of asthmatic women exhibit abnormal hormonal changes (92). These studies indicate that sex hormone fluctuations may act as a risk factor for severe asthma attacks.

Indeed, more pronounced incidence and severity of asthma starting around puberty usually occur in females but not in males (86), suggesting the important role of sex hormones in the development of female airway pathology. Estrogen is the most widely studied ovarian hormone in airway inflammation. Mouse models have suggested the role of estrogen receptor (ER) signaling in allergic airway inflammation by increasing the function of dendritic cells, M2 macrophages, and mast cells (93–95). Besides, 17β-estradiol and progesterone promote T_H_17 cell differentiation and subsequently enhance IL-17A-mediated airway inflammation (96). From the perspective of neurological factors, as the parasympathetic nerve pathway is an essential determinant of bronchial tension, estradiol can indirectly cause bronchial muscle contraction and bronchial mucosal swelling by regulating the activity of acetylcholine and cholinesterase (97, 98). Generally speaking, given the commonly harbored hormonal disorders in PCOS women, these studies provide a new insight to investigate the correlation between PCOS and asthma by elucidating the effect of sex hormones.

### Mood Disturbances

Clear evidence from epidemiological studies corroborates that women with PCOS have an increased incidence of depression, anxiety, sleep disorders, or other psychological disorders (46, 99, 100). Since the presence of overweight or obesity and infertility may exacerbate depression and anxiety reported in the general population, Damone et al. indicated that the associations between PCOS and depression or anxiety remain statistically significant, although weakened after adjusting for BMI, infertility, and sociodemographic variables (101). This confirms the hypothesis that the PCOS state itself may have an independent effect on mental functioning. When shown negative emotional images, PCOS women with IR exhibited increased activation in the left prefrontal cortex and the ventral anterior cingulate region compared with controls. These affected areas are responsible for the integration and regulation of cognitive and emotion-related information (102). In addition, PCOS women with IR were found to have greater marginal activation in emotional tasks than controls, leading to the aggravation of anxiety symptoms (103).

Meanwhile, numerous epidemiological studies have determined mental disorders as one of the important comorbidities of asthma. Adolescents with asthma are more prone to develop anxiety unipolar depressive disorder and bipolar disorder than the control group (104, 105). However, Slattery et al. have reached conflicting conclusions, demonstrating depressive symptoms were not significantly associated with a lifetime history of asthma (106). Different research methodology and diagnostic criteria, as well as a lack of adjustment for other co-existing allergic disorders, may explain the inconsistency. In recent years, magnetic resonance imaging (MRI) has been applied to investigate brain regions that may play a mechanistic role in asthma and mood disorders. Compared with the healthy controls, asthmatic patients showed abnormal structural connectivity in the bilateral frontal gyrus, right temporo-parietal cortex, and limbic regions, as well as decreased globus pallidus volume, suggesting changes in the function of brain regions involved in emotional regulation (107, 108).

Several common pathophysiological alterations may carry responsibility for mood disturbances in both PCOS and asthma, such as hypothalamic-pituitary-adrenal (HPA) dysfunction and inflammatory responses. HPA axis dysfunction is a potential mechanism underlying a variety of mental health conditions, especially anxiety and depression (109, 110). Cortisol, a steroid hormone with diurnal fluctuations, is widely recognized as a highly sensitive biomarker of stress-related changes in the maintenance of metabolic homeostasis (111). Flattening the circadian rhythm of cortisol results in dysregulation of several downstream biological and behavioral systems, including immunity, metabolism, energy, as well as appetite. Interestingly, chronic HPA axis disorders are common in asthmatic children (109). Compared with the healthy controls, asthmatic children had lower cortisol levels in hair samples, reduced cortisol reactivity in saliva samples, and a blunted cortisol awakening response (CAR) (112, 113). Further study indicated that adrenal insufficiency is not simply a consequence caused by inhaled corticosteroid treatments for asthma, it may also be a feature of asthma itself (114). Likewise, abnormal HPA activity in PCOS women has also been widely reported, but results vary. Some studies indicated that women with PCOS or different hyperandrogenic states are characterized by HPA-axis overactivity (115, 116). Nevertheless, Prelevic et al. (117) observed significantly lower cortisol levels at nighttime in PCOS women. Taken together, as abnormal cortisol levels are observed among mental disorders, asthma, and PCOS, HPA dysfunction could be an underlying mechanism that predisposes patients with asthma or PCOS to develop mood disturbances.

Inflammation responses could also explain this co-association between asthma or PCOS and multiple mental disorders. Increasing evidence shows that the levels of pro-inflammatory cytokines (e.g. IL-6, TNF-*α*, CRP) in peripheral blood of patients with mental problems are dramatically increased (118–121). Given that both asthma and PCOS are characterized by chronic systemic inflammation, these findings provide a basis for such association. The oversecreted pro-inflammatory cytokines can penetrate the blood-brain barrier during allergic reactions (122). On the one hand, it activates abnormal neuroimmune mechanisms in certain neural circuits involved in emotion regulation; On the other hand, it leads to decreased synthesis and increased uptake of 5-hydroxytryptamine, a neurotransmitter associated with various psychiatric disorders (123–125). Meanwhile, elevated levels of peripheral cytokines can activate microglia (126), which are involved in neuronal cell apoptosis, neurogenesis, and synaptic interactions (127). Inflammation-induced hyperactivation of microglia can inhibit neuronal activity through increased phagocytosis of dendritic spines, thus encouraging the development of mood disturbances (128). So far, the role of inflammatory response in the pathogenesis of mood disorders has not been fully elucidated, but it possibly becomes one of the promising targets to protect asthma or PCOS patients from mood disorders.

## Discussion

Both PCOS and asthma are common diseases in adult females, causing severe damage to women’s physical and mental health as well as their quality of life. Clarifying the full landscape of pathogenesis and development of these two diseases under physiological conditions can elucidate the complex negative effects on female health from a whole-life perspective. Previous studies mainly focus on inheritance, molecular cytology, epigenetics, or other aspects, and have already made significant breakthroughs. In recent years, growing epidemiological studies have demonstrated that PCOS, to a certain degree, correlates to asthma in several aspects. Specifically, women with PCOS are prone to develop asthma, and in return, asthma patients tend to exhibit increased susceptibility to metabolic syndrome, impaired fertility, irregular menstruation, and other clinical symptoms similar to PCOS. Furthermore, in the context of the coronavirus disease 2019 (COVID-19) pandemic, emerging data link the risk of severe COVID-19 with both PCOS and asthma. Researchers have identified potential overlap between common PCOS features and key risk factors of COVID-19, including cardio-metabolic comorbidity, hyperandrogenism, hyper-inflammation, etc. (129). Studies that have addressed the issue of asthma patients’ susceptibility to the disease have come to discrepant conclusions, possibly due to complex interplay between numerous factors such as geographical differences, asthma phenotypes, and asthma medication (130–133). Of note, patients suffering from Th2-low asthma, especially the subjects with concomitant MS, are at a higher risk for progression to severe COVID-19 (134, 135). This provides new insights into the association between PCOS and asthma and requires further research. Interestingly, PCOS and asthma have been confirmed to share common physiological and pathological changes, such as specific hormonal abnormalities, insulin resistance as well as chronic systemic inflammation, whereas the potential mechanisms linking asthma to PCOS remain poorly investigated. It is important to mention that both conditions were recognized to originate in the early stages of life (136, 137).

PCOS is considered to have a strong correlation with autoimmune and chronic systematic inflammation related to IR, T2DM, or other metabolic syndromes, suggesting a possible mechanism that may occur when it comes to airway inflammation or even asthma in PCOS women. Given that numerous studies have reported an increased incidence of asthma-related pathological changes in PCOS females, further longitudinal studies and more precise elucidation of pathogenesis are required to examine the deeper connection between PCOS and asthma. To date, most data support a negative impact of asthma on female reproductive health through inducing irregular menstruation and reduced fertility. Based on the available evidence, it could be speculated that the imbalance of sex hormones and metabolic disorders behind impaired female reproductive health is likely to be one of the potential explanations for this correlation.

Currently, most evidence comes from observational studies, making it hard to establish a causal relationship between PCOS and asthma. The pathophysiology of the two conditions may involve diverse factors, and further prospective studies are of great significance to elucidate the complex cross-influence of inflammatory, metabolic disturbance, as well as endocrine disorders, and to provide broader screening and treatment strategies for women suffering from PCOS, asthma, or related complications.

## Publisher’s Note

All claims expressed in this article are solely those of the authors and do not necessarily represent those of their affiliated organizations, or those of the publisher, the editors and the reviewers. Any product that may be evaluated in this article, or claim that may be made by its manufacturer, is not guaranteed or endorsed by the publisher.

## Author Contributions

H-FH and J-XP conceived of the study; YX and Z-YZ wrote the manuscript; J-XP and YX revised the manuscript. All authors contributed to the article and approved the submitted version.

## Funding

This work was supported by National Key R&D Program of China (2021YFC2700603), the National Nature Science Foundation of China (82088102, 82171688, 82192873), Collaborative Innovation Program of Shanghai Municipal Health Commission (2020CXJQ01), CAMS Innovation Fund for Medical Sciences (2019- I2M-5-064), Clinical Research Plan of SHDC (SHDC2020CR1008A), Shanghai Frontiers Science Center of Reproduction and Development and Research Project of Shanghai Municipal Health Commission (20204Y0234).

## Conflict of Interest

The authors declare that the research was conducted in the absence of any commercial or financial relationships that could be construed as a potential conflict of interest.

## Publisher’s Note

All claims expressed in this article are solely those of the authors and do not necessarily represent those of their affiliated organizations, or those of the publisher, the editors and the reviewers. Any product that may be evaluated in this article, or claim that may be made by its manufacturer, is not guaranteed or endorsed by the publisher.

## Abbreviations

PCOS, polycystic ovary syndrome; T2DM, diabetes mellitus type 2; CRP, C-reactive protein; AHR, hyperresponsiveness; FEV1, forced expiratory volume in one second; FVC, forced vital capacity; BMI, body mass index; MS, metabolic syndrome; IR, insulin resistance; IGT, impaired glucose tolerance; GLUT4, glucose transporter 4; IGF-1, insulin-like growth factor 1; FRC, functional residual capacity; ERV, expiratory volume; SPA, surface-active protein A; GC, granulosa cell; TTP, time to pregnancy; PTX3, pentraxin-3; PMA, perimenstrual asthma; MRI, magnetic resonance imaging; HPA, hypothalamic-pituitary-adrenal; CAR, cortisol awakening response; AMH, anti-Müllerian hormone; COVID-19, coronavirus disease 2019.
